# Intraductal Papillary Mucinous Neoplasm of the Bile Duct: A Case Report

**DOI:** 10.7759/cureus.78749

**Published:** 2025-02-08

**Authors:** Daniel Cain, Sidart Pradeep, Manal Fasih, Nicholas Shaffer, Stephen Baker, Melvin Simien

**Affiliations:** 1 Internal Medicine, Baylor Scott & White All Saints Medical Center, Fort Worth, USA; 2 Gastronenterology, Texas Christian University (TCU) Burnett School of Medicine, Fort Worth, USA; 3 Graduate Medical Education/Internal Medicine Residency, Baylor Scott & White All Saints Medical Center, Fort Worth, USA; 4 Pathology, Baylor Scott & White All Saints Medical Center, Fort Worth, USA; 5 Gastroenterology and Hepatology, Baylor Scott & White All Saints Medical Center, Fort Worth, USA

**Keywords:** bile duct neoplasms, ercp, gastroenterology, hepatobiliary neoplasms, histology, internal medicine, intrahepatic bile ducts, ipmn, ipnb, pathology

## Abstract

Intraductal papillary mucinous neoplasms (IPMNs) are a prevalent subtype of pancreatic cystic lesions, especially among individuals with liver cirrhosis. Intraductal papillary neoplasms of the bile duct (IPNBs) differ in histopathology based on the location and cellular variability in each location. Intrahepatic IPNBs are less aggressive than the extrahepatic variant, highlighting its heterogeneity and complexity.

IPNBs are largely preinvasive but can develop into invasive carcinoma. Imaging typically reveals bile duct dilation with intraductal masses but may underestimate tumor burden. Intraductal ultrasonography and cholangioscopy are recommended for assessing the tumor extent, followed by biopsy. Surgical resection is the primary treatment for nonmetastatic IPNBs, with approaches varying based on the location and extent of the tumor. Liver transplantation with duodenopancreatectomy may be necessary for incomplete resections.

This case describes a 76-year-old woman who presented with right upper quadrant pain. An abdominopelvic computed tomography (CT) revealed an ill-defined 4.4-cm mass in the left lobe of the liver. Endoscopic retrograde cholangiopancreatography (ERCP) revealed dilation in the left intrahepatic branches due to the mass, and fine-needle aspiration (FNA) showed adenomatous epithelium. Subsequent imaging identified persistent intrahepatic bile duct dilation, left lobe mass, and an abnormal porta hepatis lymph node, with FNA confirming an IPNB with low-grade dysplasia, but no metastasis to the lymph node. The patient underwent a left hepatectomy and portal lymphadenectomy. Surgical pathology confirmed IPNB without invasion. Postoperative ERCP showed no further lesions, and her cancer antigen 19-9 (CA 19-9) levels initially decreased but later began to rise. Positron emission tomography and CT imaging showed no evidence of malignancy, and she remains under continued surveillance due to persistently elevated CA 19-9 for nearly one and a half years.

In patients with mass-related intrahepatic biliary dilation, IPNB should be considered in the differential diagnosis. This case provides valuable insights into the limited literature, highlighting the complexities of diagnosing and managing IPNB, a preinvasive lesion with malignant potential. Persistent elevated CA 19-9 levels following resection underscore the need for vigilant post-resection surveillance. The case emphasizes an individualized treatment approach and calls for further research to refine surveillance protocols and improve long-term outcomes, especially in atypical presentations or incomplete resections.

## Introduction

Pancreatic cystic lesions (PCLs) have a reported prevalence of about 8% of the general population per the meta-analysis done by Zerboni et al. [1]. The mucinous subtypes of PCLs consist of intraductal papillary mucinous neoplasms (IPMNs) and mucinous cyst neoplasms. The exact incidence of IPMNs remains unclear, as improved imaging technology in recent decades has enhanced detection capabilities. A large population study conducted in Olmsted County, Minnesota, from 1984 to 2005 aimed to determine the incidence of IPMNs. The study reported an incidence of 2.04 cases per 100,000 people and a prevalence of 25.96 cases per 100,000 people at the time of completion [2]. IPMN of the biliary tract (IPMN-B) has no well-defined incidence due to its rarity, but a study of 253 histologically identified IPMNs reported a 9% incidence among the sample group [3]. The prevalence of pancreatic IPMN in adults is about 6.6%, whereas patients with liver cirrhosis have a higher prevalence of about 14%, based on a study of 192 cirrhotic patients. However, no increased incidence of IPMN-B has been reported in relation to cirrhosis, except for a single case reported by Xu et al. [4,5]. Pancreatic IPMNs and IPMN-B are more common in men than women and typically present between their fourth and sixth decades of life [6,7].

IPMN-Bs, or intraductal papillary neoplasm of the bile ducts (IPNBs), are thought to arise via similar pathways to pancreatic IPMNs, given that they have the same embryologic origin [8]. IPNB has previously been described mostly in reports from East Asia and has been associated with intrahepatic stones and/or liver flukes [9]. However, a study in the United States found IPNB in 12% of resected bile duct carcinomas, but no significant association with hepatolithiasis or liver fluke [10]. IPNBs are preinvasive lesions characterized by dilated bile ducts filled with papillary or villous biliary neoplasms surrounding the fibrovascular stalks [11]. Most pancreatic IPMNs and IPNBs are characterized by excessive intraductal mucin secretion [12,13]. IPNBs have variable intramucosal spread of neoplastic epithelia around the main tumor, with extensive spread in some cases [11]. 

Histologic staining is used to differentiate the epithelial subtypes of both pancreatic IPMNs and IPNBs to determine pancreatobiliary, intestinal, oncocytic, and gastric subtypes. The propensity for invasion and the histologic features differ among the subtypes of IPNB [11]. Most pancreatic IPMNs are of gastric or intestinal subtypes, while the pancreatobiliary subtype is more frequent in papillary bile duct lesions [10,14]. 

While location is not a significant predictor of prognosis, IPNB occurs most frequently in the hilum and left-sided bile duct system [10]. Additionally, IPNBs have been found to have different histopathologic features depending on their location in the biliary system, indicating that IPNB is possibly a heterogeneous and complex disease [11]. In terms of histologic grading, extrahepatic IPNBs have been found to almost always only have high-grade dysplasia, while intrahepatic IPNBs may have some areas of coexisting low-to-intermediate-grade dysplasia with high-grade dysplasia. Similarly, about half of intrahepatic IPNBs are invasive at the time of resection compared to the majority of extrahepatic IPNBs. Thus, intrahepatic IPNBs appear to be less aggressive than extrahepatic IPNBs [15].

## Case presentation

**Figure 1:**
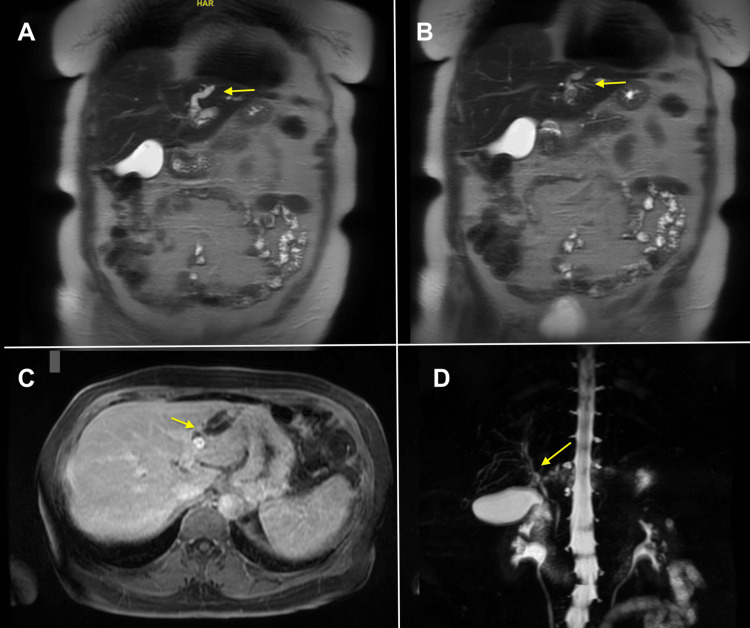
MRCP images (A, B): T2-weighted images with dilated CBD (yellow arrows). (C) Axial volumetric interpolated breath-hold examination/liver imaging with volume acceleration (VIBE/LAVA) showing intrahepatic biliary dilation (yellow arrow). (D) Coronal view demonstrating intrahepatic biliary dilation (yellow arrow). MRCP: magnetic resonance cholangiopancreatography, CBD: common bile duct.

Herein, we present a case of a 76-year-old woman with a past medical history of gastroesophageal reflux disease (GERD) treated (as needed) with omeprazole, allergic rhinitis, basal cell carcinoma, and osteopenia who was referred to gastroenterology after a computed tomography (CT) of the abdomen and pelvis, ordered by her primary care physician for right upper quadrant abdominal pain evaluation, showed an ill-defined 4.4-cm mass in the left hepatic lobe with branching hypodensities. There was concern for the hepatic mass causing intrahepatic biliary dilation, and a follow-up magnetic resonance cholangiopancreatography (MRCP) was ordered, which revealed severe lateral segment intrahepatic bile duct dilations with an ill-defined mass lateral to the falciform ligament. There were also multiple gallbladder polyps and PCLs measuring up to 1.1 cm. MRCP images are shown in Figure 1. Total bilirubin (0.4 mg/dL), alkaline phosphatase (ALP; 57 IU/L), aspartate aminotransferase (AST; 17 U/L), and alanine transaminase (ALT; 14 U/L) were all within normal limits. Carbohydrate antigen 19-9 (CA 19-9) was elevated at 5,246 U/mL, and the patient was referred to gastroenterology for further evaluation and biopsy. 

**Figure 2:**
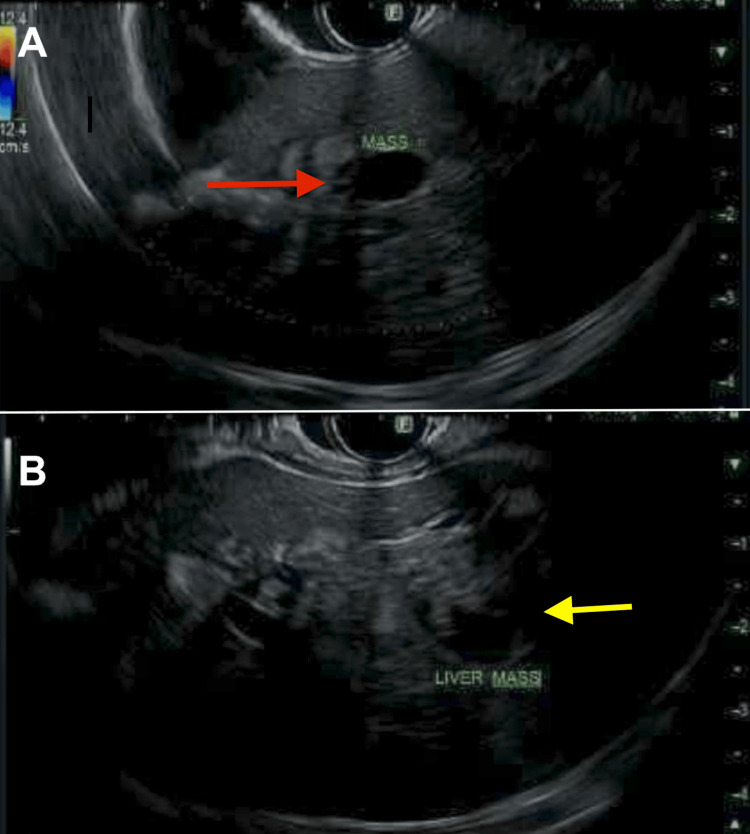
EUS images (A, B) Left hepatic hyperechoic mass seen on EUS, measuring 40 x 30 mm in maximal cross-sectional diameter (arrows). EUS: endoscopic ultrasound.

The patient underwent endoscopic retrograde cholangiopancreatography (ERCP), which revealed dilation of the left intrahepatic branches due to the mass. Biliary sphincterotomy was performed, and the biliary tree was swept. Fine-needle aspiration (FNA) was performed, which revealed fragments of adenomatous epithelium, though bile duct brushings were negative for malignancy. Samples were collected for cytological analysis, and a biliary stent was placed in the common bile duct. Figure 2 shows the images from the endoscopic ultrasound (EUS). A referral to hepatobiliary surgery was made, and concern for portal lymphadenopathy on prior imaging review was raised. A follow-up CT of the chest, abdomen, and pelvis was ordered with triple-phase contrast for further evaluation, and plans were made for a repeat ERCP with SpyGlass cholangioscopy. An EUS-guided biopsy of the portal lymph nodes was also scheduled to further evaluate the liver mass in segments two and three, where a transition point concerning cholangiocarcinoma was identified.

**Figure 3:**
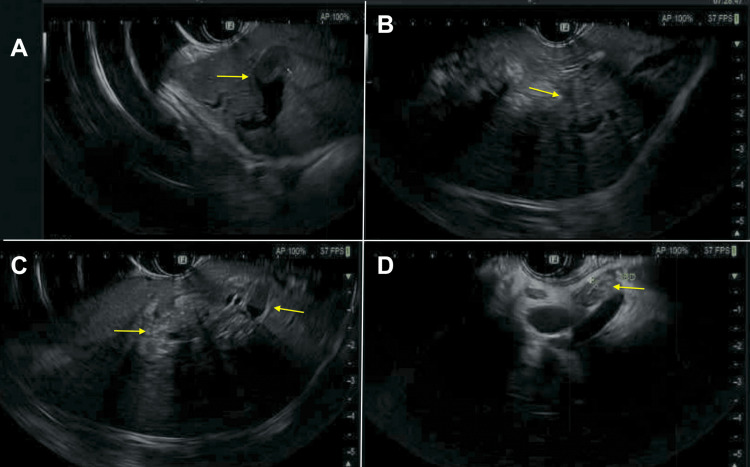
Follow-up EUS images (A-C) There is persistence in abnormal intrahepatic bile duct dilation involving the lateral greater than medial left lobes of the liver with small amounts of pneumobilia in the left liver lobe with indistinct mass centrally within the left lobe of the liver measuring approximately 2.2 x 1.8 cm (yellow arrows). (D) Common bile duct with dilation (yellow arrow). EUS: endoscopic ultrasound.

**Figure 4:**
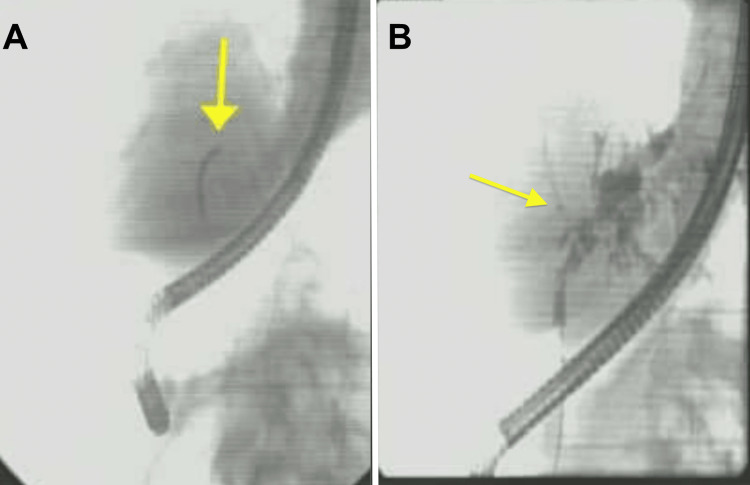
Images from the repeat ERCP with SpyGlass cholangioscopy (A) Cholangiography scope entering the biliary tree. (B) Dilated bile ducts in the left liver lobe. ERCP: endoscopic retrograde cholangiopancreatography.

**Figure 5:**
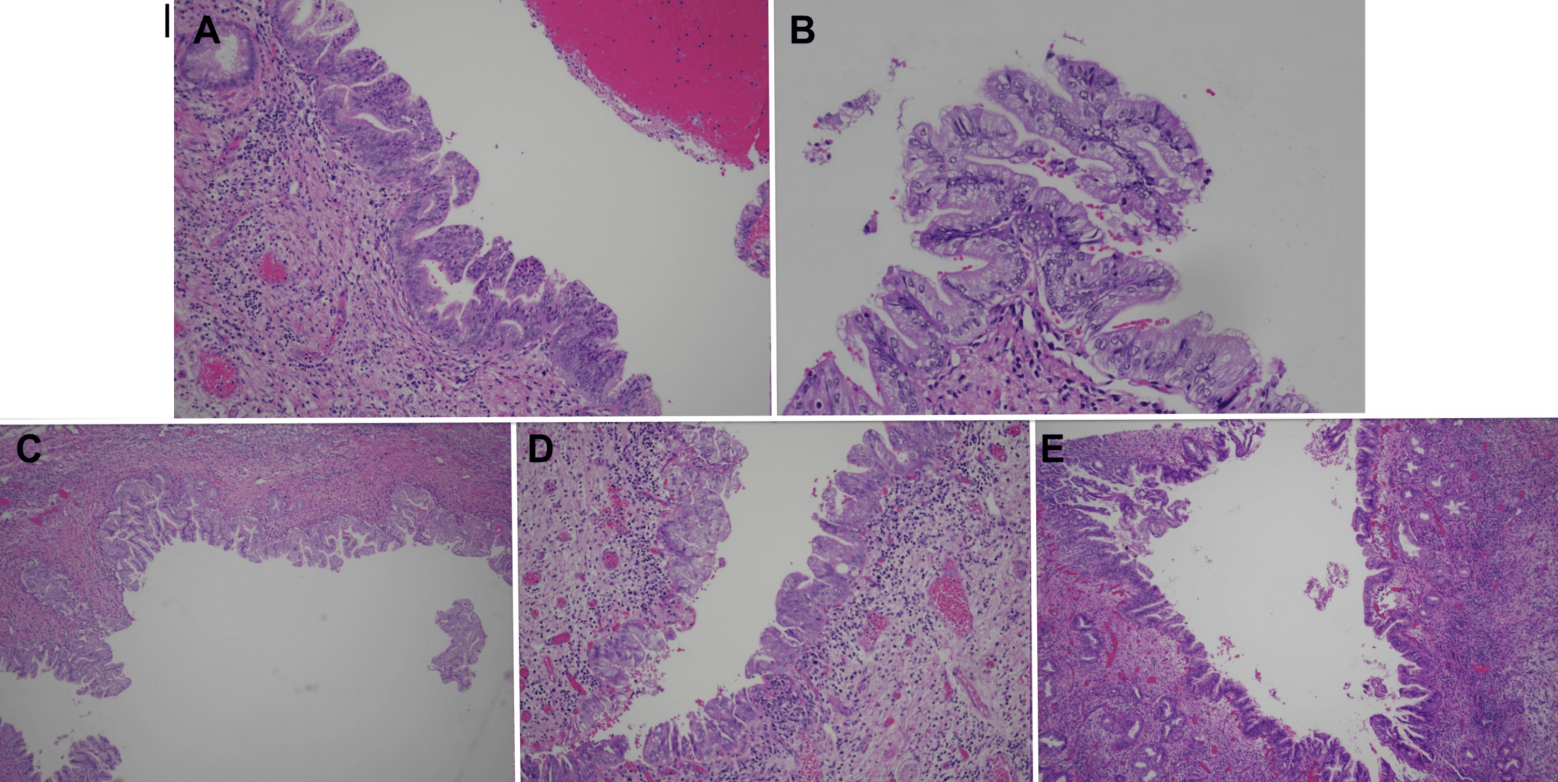
Pathology from ERCP SpyGlass cholangioscopy and partial hepatectomy (A, B) ERCP specimens with prominent biliary projections lined with mildly atypical, stratified columnar epithelium with rare mitotic features. (C-E) Partial hepatectomy specimen showing intraductal papillary neoplasm of the bile duct with low-to-intermediate-grade dysplasia, but no invasive carcinoma detected. ERCP: endoscopic retrograde cholangiopancreatography.

The follow-up EUS showed the left liver lobe mass as well as a single abnormal lymph node in the region of the porta hepatis, as shown in Figure 3. FNA was performed, which showed IPNB with low-grade dysplasia, and no evidence of malignancy in the porta hepatis lymph node. ERCP with SpyGlass cholangioscopy showed a single moderate biliary stricture, classified as Bismuth type III, in the hepatic duct system, as shown in Figure 4. A biopsy was taken from the left main hepatic duct, which showed ulcerated fragments of intraductal papillary neoplasm with prominent papillary projections lined with mildly atypical stratified columnar epithelium and rare mitotic features, consistent with IPMN-B. The patient then underwent hepatobiliary surgery for left hepatectomy and portal lymphadenectomy. Surgical pathology confirmed IPNB with low-to-intermediate-grade dysplasia, but no invasive carcinoma. Images from the pathology slides are shown in Figure 5. She was discharged after the post-op recovery period, with continued surveillance labs and imaging in the outpatient setting with no subsequent recurrence. 

## Discussion

In summary, our patient presented with right upper quadrant pain and was ultimately diagnosed with an IPNB in the left hepatic lobe, confirmed through a combination of imaging, endoscopic procedures, and histopathological studies. Surgical resection with a left hepatectomy and portal lymphadenectomy was performed, and pathology confirmed the presence of IPNB without invasion. 

IPNBs are largely preinvasive, with eventual invasion of stroma and development of invasive carcinoma [8]. The degree of intramucosal spread is variable and may be extensive [8]. Primary imaging modalities for IPNB are CT and magnetic resonance imaging (MRI). Imaging frequently reveals bile duct dilation with an associated intraductal mass [16]. On CT, lesions typically appear as an isointense or hyperintense mass during the late arterial phase, with occasional rim enhancement at the base of the lesion [16]. T1-weighted MRI reveals hypointense lesions, while T2-weighted MRI reveals hyperintense lesions [16]. However, traditional imaging modalities may underestimate tumor burden, and thus, intraductal ultrasonography and cholangioscopy should be employed to further assess the depth of invasion and tumor extent [16]. Lesions are then biopsied via percutaneous transhepatic cholangioscopy (PTCS) or peroral cholangioscopy (POCS) [9]. POCS may be preferred due to its lower risk of complications; however, in cases where tumors produce abundant amounts of mucin, PTCS allows for better characterization of the location and abundance of tumors [9].

IPMNs and IPNBs share several similarities and differences. Mucin hypersecretion is found in nearly all IPMNs and frequently in intrahepatic IPNBs; however, it is uncommon in extrahepatic IPNBs. In general, most IPNBs show high-grade dysplasia. Conversely, IPMNs show variable levels of dysplasia. For example, main pancreatic duct IPMNs usually have high-grade dysplasia, while branch duct gastric subtype IPMNs usually show low-to-intermediate-grade dysplasia. Both IPMN and IPNB eventually invade the stroma and develop into invasive adenocarcinoma; however, the respective invasive lesions usually show distinct histological differences. Invasive IPMNs are most commonly colloid carcinoma or tubular adenocarcinoma, with infrequent stromal desmoplasia. On the other hand, tubular adenocarcinoma with desmoplastic reaction is found in nearly all cases of invasive IPNB [9].

Although these tumors are benign, treatment is advisable to prevent complications such as recurrent cholangitis and obstructive jaundice [9]. The primary treatment method for nonmetastatic IPNB is via surgical resection, prior to which an assessment of tumor location and extent is important [9]. In cases where superficial tumor spread is low, transhepatic bile duct resection can be employed [9]. IPNBs involving only one of the two intrahepatic bile ducts are treated with resection of the affected hemiliver and common bile duct [17]. When the IPNB is localized to the extrapancreatic portion of the bile duct, complete bile duct resection with lymphadenectomy is recommended [17]. In cases where surgical resection is incomplete or a tumor is present on the remaining intrahepatic bile duct, liver transplantation with duodenopancreatectomy would theoretically be the only curative therapy [17].

## Conclusions

In patients with mass-related intrahepatic biliary dilatation, one should consider IPNB on the differential diagnosis. This case represents a valuable contribution to the literature, given the limited number of reported cases available for reference and clinical insights at this time. It also highlights the complexity of diagnosing and managing IPNB, a preinvasive lesion with potential for malignant transformation. IPNB, despite being preinvasive, requires close follow-up due to the risk of recurrence or progression. The persistently elevated CA 19-9 levels following resection in this case highlight the importance of continued surveillance, even when initial surgical outcomes are favorable. This case also illustrates the necessity of tailoring treatment depending on tumor location and extent. Additionally, further research is essential to refine surveillance protocols and improve long-term outcomes for patients with IPNB, especially in cases with atypical presentations or incomplete resections.
